# Application of Angiotensin Receptor–Neprilysin Inhibitor in Chronic Kidney Disease Patients: Chinese Expert Consensus

**DOI:** 10.3389/fmed.2022.877237

**Published:** 2022-07-19

**Authors:** Liangying Gan, Xiaoxi Lyu, Xiangdong Yang, Zhanzheng Zhao, Ying Tang, Yuanhan Chen, Ying Yao, Fuyuan Hong, Zhonghao Xu, Jihong Chen, Leyi Gu, Huijuan Mao, Ying Liu, Jing Sun, Zhu Zhou, Xuanyi Du, Hong Jiang, Yong Li, Ningling Sun, Xinling Liang, Li Zuo

**Affiliations:** ^1^Department of Nephrology, Peking University People's Hospital, Beijing, China; ^2^Institute of Materia Medica, Peking Union Medical College, Chinese Academy of Medical Science, Beijing, China; ^3^Qilu Hospital of Shangdong University, Qingdao, China; ^4^First Affiliated Hospital of Zhengzhou University, Zhengzhou, China; ^5^The Third Affiliated Hospital of Southern Medical University, Guangzhou, China; ^6^Guangdong Provincial People's Hospital, Guangzhou, China; ^7^Tongji Hospital Affiliated to Tongji Medical College of Huazhong University of Science and Technology, Wuhan, China; ^8^Fujian Provincial Hospital, Fuzhou, China; ^9^Bethune First Hospital of Jilin University, Changchun, China; ^10^Shenzhen Bao'an People's Hospital, Shenzhen, China; ^11^Renji Hospital Affiliated to Shanghai Jiao Tong University School of Medicine, Shanghai, China; ^12^Jiangsu Province Hospital, Nanjing, China; ^13^First Affiliated Hospital of Dalian Medical University, Dalian, China; ^14^Shandong Provincial Hospital, Jinan, China; ^15^First Affiliated Hospital of Kunming Medical University, Kunming, China; ^16^The Second Affiliated Hospital of Harbin Medical University, Harbin, China; ^17^People's Hospital of Xinjiang, Urumqi, China; ^18^Huashan Hospital, Fudan University, Shanghai, China; ^19^Peking University People's Hospital, Beijing, China

**Keywords:** chronic kidney disease, consensus, angiotensin receptor-neprilysin inhibitor, hypertension, ACEI/ARB

## Abstract

Chronic kidney disease (CKD) is a global public health problem, and cardiovascular disease is the most common cause of death in patients with CKD. The incidence and prevalence of cardiovascular events during the early stages of CKD increases significantly with a decline in renal function. More than 50% of dialysis patients die from cardiovascular disease, including coronary heart disease, heart failure, arrhythmia, and sudden cardiac death. Therefore, developing effective methods to control risk factors and improve prognosis is the primary focus during the diagnosis and treatment of CKD. For example, the SPRINT study demonstrated that CKD drugs are effective in reducing cardiovascular and cerebrovascular events by controlling blood pressure. Uncontrolled blood pressure not only increases the risk of these events but also accelerates the progression of CKD. A co-crystal complex of sacubitril, which is a neprilysin inhibitor, and valsartan, which is an angiotensin receptor blockade, has the potential to be widely used against CKD. Sacubitril inhibits neprilysin, which further reduces the degradation of natriuretic peptides and enhances the beneficial effects of the natriuretic peptide system. In contrast, valsartan alone can block the angiotensin II-1 (AT1) receptor and therefore inhibit the renin–angiotensin–aldosterone system. These two components can act synergistically to relax blood vessels, prevent and reverse cardiovascular remodeling, and promote natriuresis. Recent studies have repeatedly confirmed that the first and so far the only angiotensin receptor–neprilysin inhibitor (ARNI) sacubitril/valsartan can reduce blood pressure more effectively than renin–angiotensin system inhibitors and improve the prognosis of heart failure in patients with CKD. Here, we propose clinical recommendations based on an expert consensus to guide ARNI-based therapeutics and reduce the occurrence of cardiovascular events in patients with CKD.

## Introduction

Chronic kidney disease (CKD) is a global public health issue (1). In China, the prevalence of CKD is 7.18% and approximately 132 million patients have CKD, accounting for one fifth of all CKD patients in the world (2). CKD is usually progressive, and therefore worsens over time. In 2012, Kidney Disease: Improving Global Outcomes (KDIGO) proposed CKD staging criteria based on the estimated glomerular filtration rate (eGFR) and urinary albumin levels because eGFR levels and the urinary albumin/creatinine ratio are both correlated with poor prognosis (3).

The risk of cardiovascular events is significantly increased under CKD, with cardiovascular diseases representing the major cause of death in patients with CKD (4). Compared with the general population, the incidence and prevalence of cardiovascular events are significantly higher during the early stages of CKD (CKD stages 1–3), and the risk increases exponentially as renal function declines (5). In patients undergoing hemodialysis and peritoneal dialysis, the prevalence of cardiovascular diseases is as high as 76.5 and 65%, respectively (6). In China, over 50% of dialysis patients die of cardiovascular and cerebrovascular events (7).

Traditional risk factors such as hypertension, hyperglycemia, and dyslipidemia, as well as non-traditional risk factors such as abnormal calcium and phosphorus metabolism, in addition to inflammation, are all implicated in the high incidence of cardiovascular events in patients with CKD (4).

Hypertension is one of the most common complications in patients with CKD (8), where the control rate is poor. That is, if 130/80 mmHg is taken as the target for blood pressure control, the control rate is only 11.9% (9). Elevated blood pressure not only promotes the progression of CKD (10), but also causes myocardial remodeling in patients with CKD and increases the risk of cardiovascular events (11). Cardiovascular complications in patients with CKD include coronary heart disease, heart failure, arrhythmia, sudden death, and others. Myocardial changes observed in patients with CKD, such as early-onset left ventricular hypertrophy, significant myocardial fibrosis, and sparse capillaries, constitute the pathological basis leading to these cardiovascular events (4). The occurrence of heart failure is also related to hypertension and increased volume load, which become more difficult to control with the progression of CKD.

Generally, the blood pressure target for patients with CKD and hypertension is stricter than that for regular patients with hypertension. The SPRINT study confirmed that intensive blood pressure reduction benefited patients with CKD by alleviating cardiovascular and cerebrovascular events (12). Based on the results of the SPRINT study and a meta-analysis, the 2021 KDIGO CKD hypertension management guidelines recommended a systolic blood pressure of 120 mmHg as the optimal blood pressure reduction target (13).

Antihypertensive treatment for patients with CKD usually includes lifestyle intervention in conjunction with drug treatment (13). All five major types of antihypertensive drugs can be used for blood pressure control in patients with CKD (13). Angiotensin-converting enzyme inhibitors (ACEIs) and angiotensin receptor blockers (ARBs) not only lower blood pressure, but also exert a protective effect on target organs, as exemplified by their positive influence on renal hemodynamics and urinary albumin-reducing effects (14), which can delay the progression of CKD. As such, these drugs represent first-line treatment options for hypertension management in patients with CKD (13, 15). However, because of the complicated pathogenesis of hypertension in these patients, most require two or more antihypertension drugs at higher doses for adequate blood pressure control. Epidemiological surveys in China show that more than 60% of patients with CKD failed to achieve the blood pressure target (16, 17). In addition, ACEIs and ARBs are the first-line treatment for reducing all-cause and cardiovascular-related deaths in patients with CKD as well as heart failure with reduced ejection fraction (HFrEF) (18, 19). Renin–angiotensin–aldosterone system (RAAS) inhibitors should also be considered because of their effects on serum potassium and fluctuations in serum creatinine levels caused by hemodynamic changes, such as reduced eGFR (18). However, there is almost no evidence that RAAS inhibition improves the prognosis of patients with CKD exhibiting heart failure with preserved ejection fraction (HFpEF) (20).

Natriuretic peptides, including atrial, B-type, and C-type natriuretic peptides, are a group of polypeptides with a wide range of physiological effects, including the promotion of natriuresis and diuresis, vasodilation, RAAS inhibition, reduction of sympathetic nerve activity, and the suppression of proliferation and fibrosis (21). Sacubitril/valsartan, a compound with a co-crystal structure composed of the neprilysin inhibitor sacubitril and angiotensin receptor blocker valsartan, is the first dual angiotensin receptor and neprilysin inhibitor (22, 23). Sacubitril is metabolized into LBQ657, which is an active metabolite that effectively inhibits neprilysin, thereby suppressing the neprilysin-mediated degradation of natriuretic peptides and enhancing the beneficial effect of the natriuretic peptide system. Valsartan can block the angiotensin II-1 (AT1) receptor to inhibit RAAS. Thus, these two components can act synergistically to dilate blood vessels, prevent or reverse cardiovascular remodeling, and promote natriuresis (23).

In recent years, studies have successively confirmed that the first and so far the only angiotensin receptor–neprilysin inhibitor (ARNI) sacubitril/valsartan can reduce blood pressure in patients with CKD more effectively than RAAS inhibitors and can improve the prognosis in patients with heart failure (24). Considering current approaches to the diagnosis and treatment of CKD accompanied by hypertension and heart failure, as well as the need to better guide the application of ARNI in patients with CKD, we formulate a consensus based on clinical evidence and experience. In this consensus, ARNI refers specifically to sacubitril/valsartan. The strength of recommendation in this consensus is indicated as Level 1 or Level 2, the quality of supporting evidence is shown as A, B, C, or D following KDIGO guidelines (13).

## Effects of CKD on the *In vivo* Metabolism of ARNI, Dose Adjustment, and Drug Interactions

### Is It Necessary to Adjust the Dosage of ARNI for Patients With Hypoproteinemia?

**Suggestion 2.1:** When ARNI is used for patients with hypoproteinemia (serum albumin <25 g/L), titration should be started from a low dose. The dosage and frequency that is suitable for patients should then fall within a safe-dose range (see Suggestion 2.2 for hypoproteinemia caused by hepatic dysfunction). *(2D)*

**Rationale:** ARNI has a high protein binding rate (16, 25). The protein binding rates of sacubitril, valsartan, and sacubitril metabolite LBQ657 are 97, 96, and 97%, respectively (25). Therefore, under hypoproteinemia, the conventional dose may result in a higher free drug concentration and enhanced efficacy of ARNI, in turn leading to side effects such as hypotension, diuresis, electrolyte disturbance, and a rapid increase in serum creatinine levels. Further, the apparent distribution volume and drug clearance of LBQ657 and valsartan increase under hypoproteinemia. As drugs are excreted faster, the frequency of medication should be adjusted accordingly.

### Adjusting the Dose for Patients With Hepatic Dysfunction

**Suggestion 2.2:** For patients with mild hepatic impairment (Child–Pugh A), there is no need to adjust the loading dose, whereas in cases of moderate hepatic impairment (Child–Pugh B), the loading dose should be halved and the dose should be gradually increased to the most suitable dose that the patient can tolerate. ARNI is not recommended for patients with severe hepatic impairment. *(1B)*

**Rationale:** A pharmacokinetic study in patients with mild to moderate hepatic impairment revealed that, compared with healthy subjects, exposure to sacubitril, LBQ657, and valsartan was slightly increased in patients with mild hepatic impairment (by 1.5, 1.5, and 1.2 times, respectively). In patients with moderate hepatic impairment, the exposure increased by 3.4, 1.9, and 2.1 times, respectively (16). In another clinical trial involving 32 patients with mild to moderate hepatic dysfunction, the AUC of sacubitril increased by 53–245%, that of LBQ657 increased by 48–90%, and that of valsartan increased by 19–109%. Further, the C_max_ of LBQ657 and valsartan were not significantly different between patients with mild and moderate hepatic dysfunction (26). Therefore, we suggest that there is no need to adjust the loading dose in patients with mild hepatic impairment; however, the loading dose should be halved in those with moderate hepatic impairment. The above liver function staging refers to Child-Pugh staging, see Supplementary Table 1 for details. At present, there are no pharmacokinetic data for patients with severe hepatic impairment, biliary cirrhosis, or cholestasis; thus, the use of ARNI is not recommended in the case of severe hepatic impairment (16).

### Use of ARNI in Patients With Heart Failure

#### ARNI in Patients With Chronic Heart Failure

**Suggestion 2.3.1:** In patients with chronic heart failure, we suggest beginning with a low dose then gradually increasing the dosage. In patients who have not previously taken ACEIs or ARBs, the recommended loading dose is 50 mg twice-daily (BID). Thereafter, depending on the patient's tolerance, the dose should be doubled every 2–4 weeks until the target dose of 200 mg BID is reached. For patients who have previously used ARBs, we suggest converting to the appropriate ARNI dose according to the previous ARB dose. For patients who have used ACEIs, it is necessary to discontinue the drug for more than 36 h before converting to an appropriate ARNI dose with reference to the original ACEI dose.

**Rationale:** For patients with heart failure, it is usually appropriate to start ARNI treatment at a relatively low dose to improve tolerance to the initial treatment. The target dose of 200 mg BID is based on previous studies, which showed that 200 mg ARNI can fully inhibit neprilysin and enhance the activity of natriuretic peptides (27, 28). In the PARADIGM-HF study, ARNI was administered to patients with HFrEF, starting with 100 mg BID and gradually increasing to 200 mg BID. By the end of the study, 76% of the patients could still maintain the target dose, which indicated that this dosage regimen was well tolerated (29). The same dosage regimen of ARNI was also adopted for HFpEF patients in the PARAGON-HF study. By the end of the study, although no significant benefit was seen for the primary endpoint in the overall HFpEF population, 82% of the patients could maintain the target dose, which indicated that this regimen was also well tolerated (30).

#### How Long After Acute Heart Failure Can ARNI Be Administered?

**Suggestion 2.3.2:** In patients with acute heart failure, ARNI treatment should be initiated from a low dose immediately after reaching hemodynamic stability. The criteria for hemodynamic stability are shown in Supplementary Table 2. *(1A)*

**Rationale:** The PIONEER-HF and TRANSITION studies evaluated the benefits of initial ARNI treatment after stabilization of acute heart failure (31, 32). The PIONEER-HF study showed that starting ARNI directly after reaching hemodynamic stability in patients with acute heart failure significantly reduced the risk of composite clinical endpoints (death, rehospitalization owing to heart failure, and left ventricular assist device implantation) by 31% (*P* < 0.002) (32). Analysis of a high-risk subgroup in the PIONEER-HF study (Supplementary Tables 3, 4) indicated that the benefits of ARNI in reducing cardiovascular death or rehospitalization owing to heart failure were comparable to those of ACEI treatment in the high-risk subgroup. Further, the risks of adverse reactions, including the deterioration of renal function, symptomatic hypotension, and hyperkalemia, were similar between high-risk and low-risk patients (33). The TRANSITION study demonstrated that, in patients with HFrEF who required hospitalization because of acute decompensated heart failure, the early initiation of ARNI treatment after reaching hemodynamic stability during hospitalization reduced the rehospitalization rate after discharge, with nearly half of the patients reaching the target dose within 10 weeks. ARNI initiation before discharge was well tolerated, and relatively few patients permanently discontinued the drug because of adverse reactions (31). Therefore, for patients with hemodynamic stability, ARNI should be initiated as early as possible with dose titration.

### Are There Any Drug Interactions or Other Matters Requiring Attention When Using ARNI in Combination With Other Agents?

#### Can ARNI Be Used With ACEI?

**Suggestion 2.4.1:** Concomitant use of ARNI and ACEI should be avoided (Table 1), and it is recommended to wait 36 h after ACEI is discontinued before using ARNI. *(1C)*

**Table 1:** Conditions and reasons not treated with ARNI.

**Conditions and reasons not treated with ARNI**
Concomitant use with ACEI: may increase the risk of angioedema
In patients with a previous history of angioedema: may increase the risk of angioedema
Concomitant use with aliskiren: may increase the risk of adverse events
In pregnant women: may cause fetal injury

*ARNI, angiotensin receptor–neprilysin inhibitor*.

**Rationale:** The concomitant use of ACEI while inhibiting neprilysin may increase the risk of angioedema (16). The development of a compound preparation comprising an neprilysin inhibitor and ACEI was previously halted because of severe angioedema and a significantly increased mortality rate (34). Therefore, it is recommended to initiate ARNI 36 h after the last dose of ACEI (16).

#### Can ARNI Be Used With SGLT2i?

**Suggestion 2.4.2:** ARNI can be used with SGLT2i in patients with heart failure. When used with diuretics, the dose should be adjusted. *(2B)*

**Rationale:** The mechanisms of action for ARNI and SGLT2i are relatively clear. ARNI acts on the natriuretic peptide system and RAAS, which blocks the adverse effects of the latter, while also enhancing the beneficial effects of the former, thereby playing a role in reversing cardiac remodeling and improving heart failure (35). SGLT2i can increase urine glucose excretion, natriuresis, and osmotic diuresis, and can act on myocardial cells to improve cardiac function (36). As ARNI and SGLT2i act through distinct mechanisms without overlap, they can be used in combination. In a randomized controlled study of SGLT-2i dapagliflozin (DAPA-HF study), 11% of patients used the combination with ARNI, which was shown to be safe and effective (37). Therefore, the two drugs can be used together by patients with heart failure. In a multicenter observational study, adding ARNI to SGLT2i for T2DM patients with HFrEF afforded better protection against renal function (38). Moreover, a study evaluated the safety and efficacy of the ARNI+SGLT2i combination in patients with HFrEF and diabetes, indicated that ARNI+SGLT2i improved the clinical and renal prognosis more significantly than ACEI/ARB+other hypoglycemic drug regimens (39). Moreover, combining SGLT2i with ARNI further reduced the risk of hospitalization owing to heart failure as well as that of major composite endpoints (hospitalization owing to heart failure or all-cause death). The combination was well tolerated, and the risks of creatinine elevation and hyperkalemia were lower than those for ACEI/ARB (39). However, in clinical practice, attention should be paid to the increased risk of hypovolemia when combining ARNI with SGLT2i. In the DAPA-HF study, SGLT2i combined with ARNI resulted in hypovolemia in 10.8% of patients (37). When used in combination, diuretics may synergistically act on nephrons with ARNI, mineralocorticoid receptor antagonists (MRA), and SGLT-2i to enhance the effects on natriuresis and diuresis. Therefore, it is necessary to adjust the doses of diuretics in time, then adjust the ARNI and SGLT-2i dosage as necessary.

#### Incompatibilities and Precautions for Concomitant Use With Other Drugs

**Suggestion 2.4.3.1:** ARNI is contraindicated in patients with a previous history of angioedema (Table 1). *(1C)*

**Rationale:** Patients with a previous history of angioedema may have an increased risk of angioedema during ARNI treatment (16), which is related to the reduced inactivation of bradykinin and substance P (40). Therefore, ARNI should not be used in patients with a known history of angioedema associated with ACEI/ARB treatment or hereditary angioedema (37). In the PARADIGM-HF study, 0.5% of patients treated with ARNI developed angioedema (37). Angioedema limited to the face and lips can generally be relieved without treatment, and antihistamines can also be applied to help relieve symptoms. Angioedema with laryngeal edema may endanger the life of patients; therefore, timely and appropriate treatment is required to keep the airway unobstructed (16).

**Suggestion 2.4.3.2:** ARNI in combination with aliskiren is not recommended (Table 1). *(1C)*

**Rationale:** Aliskiren is a direct inhibitor of renin, and its combination with ACEI or ARB may cause dual inhibition of the renin–angiotensin system and increase the risk of poor prognosis (41). According to several randomized controlled trials using aliskiren conducted by the European Medicines Agency, aliskiren used in combination with ACEI/ARB, especially in patients with impaired renal function, increased the risk of adverse events (e.g., hypotension, syncope, hyperkalemia, and acute renal failure) (42). Therefore, we suggest avoiding the use of aliskiren combined with ACEI or ARB. Although there is currently no evidence related to the combination of ARNI and aliskiren, from a mechanistic perspective, ARNI combined with aliskiren may also lead to dual inhibition of the renin–angiotensin system and increase the risk of adverse reactions; therefore, their combined use is also not recommended (16).

**Suggestion 2.4.3.3:** ARNI combined with spironolactone, amiloride, or potassium salt increases the risk of hyperkalemia (*1A*).

**Rationale:** ARNI may induce hyperkalemia. In the PARADIGM-HF study, 12% of patients receiving ARNI and 14% of those receiving enalapril developed hyperkalemia. Thus, the concomitant use of spironolactone, amiloride, or potassium salt with ARNI may increase potassium levels in the blood. In severe cases, hyperkalemia can lead to renal failure, muscular paralysis, arrhythmia, and cardiac arrest. Therefore, when using ARNI in combination with any of these three agents, close attention should be paid to serum potassium levels to avoid hyperkalemia.

**Suggestion 2.4.3.4:** ARNI is contraindicated in pregnant women to avoid the risk of teratogenicity at the early stage as well as the inconvenience and risk of subsequent drug adjustment (Table 1). *(2C)*

**Rationale:** The application of ARNI in pregnant women may cause fetal injury. Further, the application of drugs that inhibit the RAAS in the second and third trimesters may affect fetal renal function and increase the risk of illness and death of the fetus and neonate (16). In animal studies, the use of ARNI in rats and rabbits during organogenesis led to an increase in embryo-fetal mortality, and teratogenicity was observed in rabbits (16). Therefore, ARNI is not recommended for use in pregnant women in order to avoid the risk of early-stage teratogenicity as well as the inconvenience and risk of subsequent drug adjustment.

### Will the Efficacy of ARNI Be Affected if Taken With a Meal?

**Suggestion 2.5:** ARNI intake with food has no effect on its clinical efficacy. *(2B)*

**Rationale:** When ARNI was taken with a meal, exposure to sacubitril and valsartan was significantly lower, whereas exposure to the active metabolite LBQ657 exhibited no significant change (43). Although both sacubitril and valsartan exposure decreased, this decrease did not result in a clinically significant decrease in efficacy (16, 43). Therefore, ARNI can be taken with or without a meal (16, 43).

## Use of ARNI in Non-Dialysis Patients with CKD

### What Are the Indications of ARNI in Non-dialysis Patients With CKD?

**Suggestion 3.1.1:** ARNI is recommended for non-dialysis patients with CKD and heart failure. *(1A)*

**Rationale:** The efficacy of ARNI in non-dialysis patients with CKD and heart failure has been confirmed by several studies. Analysis of the CKD subgroup in the PARADIGM-HF study (eGFR: 30–60 mL/min/1.73 m^2^) revealed that ARNI reduced the risk of cardiovascular events compared with the ACEI-treatment group, with the risk of cardiovascular death decreasing by 24%, the risk of hospitalization owing to heart failure and all-cause death decreasing by 21%, and the risk of preset composite kidney events was reduced by 36% (the first occurrence of eGFR decreasing by 50% compared to the patient's baseline, decreasing from >30 to <60 mL/min/1.73 m^2^, or progressing to end-stage renal disease) (29, 44). At the same time, UACR was slightly increased in the ARNI treatment group accompanied by a delay in the decline of eGFR, suggesting that improved renal perfusion due to improved cardiac function may be the reason for the increase of UACR (44). A real-world study in Taiwan also showed that, in patients with CKD and HFrEF with different eGFR values (including 102 patients with GFR <30 mL/min/1.73 m^2^), ARNI treatment significantly reduced the risk of cardiovascular death and hospitalization owing to heart failure (45). In addition, a real-world study in Italy also showed that ARNI treatment for 12 months significantly increased eGFR in patients with CKD from the baseline (*P* = 0.011) (46). In patients with CKD exhibiting HFpEF, analysis of the CKD subgroup (eGFR: 30–60 mL/min/1.73 m^2^) in the PARAGON-HF study showed that ARNI significantly reduced the risk of the renal composite endpoint by 50% compared with ARB (30). A recent retrospective cohort study in Taiwan showed that, in patients with CKD exhibiting HFpEF (average eGFR: 70.9 mL/min/1.73 m^2^), the eGFR under ARNI treatment was significantly higher than that under valsartan treatment (*P* < 0.01) (47). Further, a recent meta-analysis revealed that ARNI significantly improved eGFR and reduced the level of NT-proBNP compared with ACEI/ARBs (48).

**Suggestion 3.1.2:** ARNI is recommended for non-dialysis patients with CKD exhibiting hypertension. *(1B)*

**Rationale:** ARNI can simultaneously act on the RAAS and natriuretic peptide system, thereby reducing blood pressure via multiple mechanisms and protecting target organs (49). More than 10 randomized controlled studies have confirmed the antihypertensive efficacy of ARNI (50). A meta-analysis involving five randomized controlled studies compared the efficacy of ARNI and ARB in patients with hypertension. After 12 weeks of treatment, ARNI more significantly reduced the mean sitting systolic blood pressure (SBP), mean sitting diastolic blood pressure (DBP), mean ambulatory SBP, and mean ambulatory DBP by 5.41 mmHg, 1.22 mmHg, 4.58 mmHg, and 2.17 mmHg, respectively, than ARB (olmesartan or valsartan) (51). The efficacy of ARNI in patients with CKD exhibiting hypertension has also been confirmed. In a multicenter, open-label study in Japan, 32 patients with CKD exhibiting hypertension were included; after ARNI treatment for 8 weeks, their average SBP and DBP in the sitting position decreased by 20.5 ± 11.3 and 8.3 ± 6.3 mmHg, respectively (52). The PARAMETER study showed that ARNI could control nocturnal blood pressure better than olmesartan. Further, as nocturnal hypertension is common in patients with CKD, ARNI could be the preferred treatment for patients with CKD exhibiting hypertension (53).

### When ARNI Is Used in Patients With Renal Impairment, How Should the Dose Titration Be Performed?

**Suggestion 3.2.1:** In patients with heart failure accompanied by mild renal impairment, there is no need to reduce the dose. In those with moderate or severe renal impairment, 25–50 mg BID is recommended as the loading dose (depending on the patient's blood pressure). If the patient can tolerate this dose, the dose should be doubled every 2–4 weeks until reaching the target maintenance dose of 200 mg BID (Table 2). *(2B)*

**Table 2:** Dosage recommendations for ARNI in CKD.

**Conditions treated with ARNI**			**Initial doses**	**Target doses**
Patients with non-dialysis CKD	Heart failure	eGFR≥60 mL/min/1.73 m^2^	50 mg BID	200 mg BID
		eGFR <60 mL/min/1.73 m^2^	25–50 mg BID	
	Hypertension	eGFR≥60 mL/min/1.73 m^2^	200 mg QD or 100 mg BID	400 mg QD or 200 mg BID
		eGFR 15-60 mL/min/1.73 m^2^	100 mg QD or 50 mg BID	
Patients with CKD undergoing maintenance dialysis: heart failure or hypertension			25–50 mg QD	100–150 mg QD to BID

*ARNI, angiotensin receptor–neprilysin inhibitor; eGFR, estimated glomerular filtration rate; QD, once-daily; BID, twice-daily*.

**Rationale:** In patients with renal impairment, exposure to sacubitril active metabolite LBQ657 was affected, whereas exposure to sacubitril and valsartan was not affected, and the difference in LBQ657 exposure was not significant under mild renal impairment. However, in patients with moderate, severe, and end-stage renal disease (non-dialysis), the exposure increased by 2.29, 2.90, and 3.27 times, respectively (54). Furthermore, the LBQ657 half-life was prolonged from 12 h to 21.1 h, 23.7 h, and 38.5 h, respectively, in patients with mild, moderate, and severe renal impairment (54). Therefore, it is not necessary to adjust the dose of ARNI for patients with mild renal impairment. For those with moderate and severe renal impairment, it is recommended to start with 25–50 mg BID and gradually increase the dose (16). The diagnosis criteria for mild, moderate, and severe renal impairment are shown in Supplementary Table 5.

**Suggestion 3.2.2:** When used in patients with essential hypertension, the conventional loading dose is 200 mg once-daily (QD). For patients whose blood pressure cannot be sufficiently controlled via a single administration daily, the dose can be increased to 400 mg QD *(1B)*. In patients with CKD, a twice-daily dosage regimen can also be adopted *(2C)*. For patients with mild renal impairment (eGFR 60–90 mL/min/1.73 m^2^), there is no need to adjust the dose (1B), whereas for those with moderate and severe renal impairment (eGFR 15–60 mL/min/1.73 m^2^), it is recommended to reduce the loading dose to 100mg QD (Table 2) *(2C)*. However, considering the minimal experience of patients with end-stage renal disease, these clinical findings should also be considered in this case *(2C)*.

**Rationale:** In terms of pharmacokinetics, the half-lives of LBQ657 and valsartan after a single dose of ARNI 200 mg are 11.5 h and 9.9 h (55). Thus, once-daily administration can be adopted. The choice of 200 mg or 400 mg QD for patients with hypertension is based on two Phase II dose-finding studies, which showed that these dosages could improve ambulatory blood pressure and office blood pressure more effectively than the control treatment, while being safe and tolerable (28, 56). In several randomized control trials on ARNI for the treatment of hypertension, once-daily administration was adopted, and the results indicated that ARNI was more effective at lowering blood pressure, including ambulatory blood pressure and nocturnal blood pressure, than the control conditions (53, 57–60). Among patients with renal impairment, mild impairment did not affect exposure to the main components and metabolites of ARNI, whereas in patients with moderate and severe renal impairment, exposure to LBQ657 was significantly increased, by 2–3 times. Therefore, for patients with moderate and severe renal impairment, we suggest adopting a low loading dose then gradually increasing to a suitable target dose (54). In a study from Japan, patients with CKD and hypertension (eGFR15–60 mL/min/1.73m^**2**^) were treated with ARNI 100–400 mg QD for 8 weeks according to their blood pressure. At the end of the study, 26 patients received LCZ696 at 200 mg QD, whereas 18 patients received LCZ696 at 400 mg QD. During the eight-week treatment period, no serious adverse reactions were noted. These results indicated that the regimen was generally well tolerated, with no adverse events such as hypotension and angioedema (52). At present, there is no medical data on patients with end-stage renal disease and eGFR <15 mL/min/1.73 m^2^. Thus, it is necessary to evaluate the benefits and risks in these patients based on clinical observations. Currently, there is no evidence as to whether BID administration of ARNI can further improve nocturnal blood pressure compared to once-daily administration. However, patients with CKD often suffer from nocturnal hypertension. Thus, a twice-daily regimen can also be used in these patients if necessary.

### What Possible Side Effects Should Be Considered During Titration of the ARNI Dose?

#### Hypovolemia and Hypotension

**Suggestion 3.3.1:** For patients with symptomatic hypovolemia, the blood pressure should be closely monitored. For patients with SBP <100 mmHg or hypotension symptoms, we recommend first adjusting the doses of diuretics and combined antihypertensive medication, treating other causes of hypotension, and correcting hypovolemia via oral or intravenous rehydration. *(1C)*

**Rationale:** In the PARADIGM-HF study, 14% of patients treated with ARNI developed symptomatic hypotension (SBP <100 mmHg), which was slightly higher than the proportion in the enalapril group (9.2%) (29). In a meta-analysis of three studies comparing ARNI and ACEI/ARB therapies, although the incidence of hypotension in patients receiving ARNI was slightly higher than that in those receiving ACEI/ARB, there was no statistically significant difference (48). We suggest that hypovolemia should be corrected before initiating ARNI, or a low loading dose should be used to reduce the risk of hypotension. If hypotension occurs during treatment, we suggest first adjusting the doses of diuretics and combined antihypertensive drugs, then treating other causes of hypotension (16). If hypotension persists after taking the above measures, dose reduction or temporary discontinuation of ARNI may be considered (16). Usually, there is no need to permanently discontinue treatment.

#### Hyperkalemia and Other Electrolyte Disorders

**Suggestion 3.3.2:** For patients with hyperkalemia (≥5.0 mmol/L), we recommend stopping potassium supplementation and MRA, and lowering potassium. If necessary, dose reduction or discontinuation of ARNI may be considered. *(1B)*

**Rationale:** ARNI treatment may lead to an increase in serum potassium because of its effect on the RAAS (16). In the PARADIGM-HF study, 12.3% of patients treated with ARNI reported hyperkalemia (serum potassium >5 mmol/L); the incidence was slightly lower than that after treatment with enalapril (13.5%) (61). In the UK HARP-III study, the incidence of hyperkalemia in patients with CKD treated with ARNI or irbesartan (GFR: 20–60 mL/min/1.73 m^2^) was similar (32 vs. 24%, *P* = 0.10) (62). For patients with hyperkalemia, priority should be given to controlling the risk factors that may lead to high potassium, potassium supplementation and MRA should be discontinued, potassium-lowering drugs should be used as necessary, and down-titration or discontinuation of ARNI should be considered according to the situation (16). The serum potassium levels of patients should be closely monitored, and ARNI treatment should be gradually resumed after the serum potassium level returns to normal (<5.0 mmol/L).

#### Rapid Decline of Renal Function

**Suggestion 3.3.3:** There are no available clinical trial data regarding the respective evaluation criteria or treatment methods for patients exhibiting a rapid decline in renal function resulting from ARNI treatment. Thus, treatment methods for renal function decline due to RAAS inhibitor treatment can be referred to instead. First, it is necessary to clarify the reasons for the decline of renal function. After excluding renal artery stenosis as the culprit, if the serum creatinine levels increase by <30% compared with the baseline value, ARNI can be used continuously. If serum creatinine levels exceed the baseline level by 30%, the dose should be reduced or ARNI should be discontinued in time, and the underlying reasons should be identified. If serum creatinine levels exceed the baseline level by 50%, it is recommended to discontinue ARNI. *(1C)*

**Rationale:** Because of its effect on the RAAS, ARNI treatment may lead to an increase in creatinine. In the PARADIGM-HF study, 5% of patients treated with ARNI reported renal failure (63). For patients exhibiting a rapid decline of renal function, we suggest correcting/excluding other potential culprits (e.g., drugs affecting renal function, hypovolemia, or urinary tract infection), then considering down-titration of ARNI according to the situation. If the patient's serum creatinine increases by <30% compared with the baseline value, ARNI can be used continuously. If the serum creatinine level exceeds the baseline level by 30%, the dose should be reduced or ARNI should be discontinued in time, and the reasons should be identified (64).

#### Dose Adjustment of Hypoglycemic Drugs

**Suggestion 3.3.4.:** In patients with Stage 3–5 CKD, doses of hypoglycemic drugs should be adjusted according to their renal function. When ARNI is combined with common oral hypoglycemic drugs, no clinically significant drug interaction is observed; however, ARNI can improve blood glucose control. Therefore, we suggest monitoring blood glucose levels and appropriately adjusting the dose of insulin secretagogue or insulin. *(2B)*

**Rationale:** When patients with diabetes exhibit renal insufficiency, renal gluconeogenesis is weakened and the clearance rates of some drugs are reduced, which may in turn increase the risk of hypoglycemia. In addition, some hypoglycemic drugs or their metabolites can accumulate in the kidneys, which further aggravates renal insufficiency. Therefore, it is necessary to select appropriate hypoglycemic drugs and dosage based on the renal function of patients with CKD (65). At present, it has only been observed that ARNI combined with metformin might cause a slight decrease in metformin exposure, with no effect on clinical efficacy. Thus, there is no need to adjust the dose of metformin. To date, no drug interaction between ANRI and other hypoglycemic drugs has been reported (16). Some studies have shown that ACEI-enkephalinase inhibitor treatment could improve insulin-mediated glucose processing, induce insulin sensitization, and improve blood glucose control (66, 67). Therefore, when ARNI is used in combination with hypoglycemic drugs, we suggest closely monitoring blood glucose and appropriately reducing the dose of insulin secretagogue or insulin in patients with a high risk of hypoglycemia to reduce this risk.

### Who Should Switch to ARNI Among Patients Using ACEIs/ARBs?

#### For Patients With Heart Failure, We Recommend Switching to ARNI

**Suggestion 3.4.1:** For patients with CKD exhibiting heart failure, ACEI/ARB can be switched to ARNI, as long as there is no contraindication, to improve eGFR, reverse cardiac remodeling, and reduce the risk of end-stage renal disease and cardiovascular events. *(1A)*

**Rationale:** The PARADIGM-HF study showed that, compared with ACEI, ARNI significantly delayed the decline rate of eGFR in patients with HFrEF by 23.5%, and reduced the risk of cardiac and renal adverse events in patients with CKD and HFrEF (29). The PARAGON study confirmed that, compared with ARB, ARNI significantly reduced the risk of adverse renal outcomes in patients with HFpEF and eGFR <60 mL/min/1.73 m^2^ (30). The EVALUATE-HF study showed that, compared with enalapril, ARNI rapidly reversed cardiac remodeling after 3 months of treatment. The UK-HARP-III study revealed that, compared with ARB, ARNI could significantly improve NT-proBNP and troponin levels, while effectively reducing blood pressure and proteinuria in patients with CKD. A meta-analysis including 3,640 patients with heart failure and CKD from three randomized controlled trials (HARP-III, PARADIGM-HF, and PARAMOUNT) indicated that, compared with ACEI/ARB, ARNI significantly improved eGFR and decreased blood pressure and NT-proBNP levels (48).

#### If Blood Pressure Is Not Well Controlled, We Recommend Switching to ARNI

**Suggestion 3.4.2.1:** For patients with CKD and poor blood pressure control, after a whole day of treatment with ACEI/ARB, we suggest switching ACEI/ARB to ARNI to further improve blood pressure control. *(1B)*

**Rationale:** A meta-analysis including five randomized controlled studies confirmed that, compared with ACEI/ARB, ARNI more effectively reduced the mean sitting arterial pressure (weighted mean difference: −5.41 mmHg, *P* < 0.01) and diastolic pressure (weighted mean difference: −1.22 mmHg, *P* < 0.01) (68). An eight-week multicenter, open-label study showed that ARNI treatment for 1 week reduced the office SBP, DBP, and pulse pressure of patients with severe hypertension by 18.7, 10.3, and 8.3 mmHg, respectively, with a more significant decrease observed in the 8 week (69). A meta-analysis including 6,064 patients with hypertension from 12 studies, of which four studies compared the antihypertensive effects of 400 mg ARNI and ARB for 8 weeks, revealed that ARNI caused a more significant reduction in 24 h ambulatory SBP (by 4.31 mmHg) and ambulatory DBP (by 1.69 mmHg) than ARB (50).

**Suggestion 3.4.2.2:** For patients with CKD and poor nocturnal blood pressure control treated with ACEI/ARB, we suggest switching ACEI/ARB to ARNI to further improve nocturnal blood pressure control. *(1B)*

**Rationale:** A randomized controlled study in patients with hypertension in Asia showed that ARNI treatment for 8 weeks effectively reduces nocturnal ambulatory SBP by up to 16.14 mmHg (56). Another randomized, double-blind, placebo-controlled, and active drug-controlled study including 1,328 patients with hypertension showed that, compared with ARB, the nocturnal mean sitting SBP of patients treated with ARNI decreased significantly by up to 9.01 mmHg (28). A different randomized controlled study revealed that, compared with olmesartan, ARNI further reduced the nocturnal mean ambulatory SBP of elderly patients with hypertension by 5.9 mmHg (53).

#### For Patients With Heart Failure Whose Serum Potassium Tends to Increase When Using ACEIs/ARBs, We Suggest Switching to ARNI After Serum Potassium Decreases to the Normal Range

**Suggestion 3.4.3:** For patients with CKD and heart failure, and whose serum potassium tends to increase when using ACEIs/ARBs, we recommend switching to ARNI after serum potassium levels decrease to the normal range so as to reduce the risk of hyperkalemia. *(2B)*

**Rationale:** The use of ACEI/ARB will decrease potassium excretion through the kidneys and increase serum potassium levels. In the PARADIGM-HF study (which excluded patients with eGFR <30 mL/min/1.73 m^2^), the serum potassium level was lower in the ARNI treatment group than in the enalapril group, and the incidence of hyperkalemia and severe hyperkalemia was significantly reduced, with even patients treated in combination with MRA exhibiting a significantly lower risk of hyperkalemia (61, 70). Moreover, the incidence of hyperkalemia was significantly lower in the ARNI group than in the valsartan group (13.2 vs. 15.3%, *P* = 0.048) (30). A meta-analysis of 11 randomized controlled trials comparing ARNI with RAAS inhibitors showed that, compared with those receiving ACEI/ARB, the ARNI group had a slightly lower risk of hyperkalemia (RR = 0.95; 95% CI = 0.88–1.02) (71). A meta-analysis published in ASN in 2020 showed similar results (72); however, patients with heart failure were mainly included in these studies, whereas patients with eGFR <30 mL/min/1.73 m^2^ or serum creatinine level > 221 μmol were excluded. To data, there are no data on patients without heart failure.

#### For Patients With Heart Failure Whose Serum Creatinine Levels Rise Sharply, We Suggest Discontinuing ACEI/ARB and Switching to ARNI

**Suggestion 3.4.4:** For patients with CKD exhibiting heart failure who are treated with ACEI/ARB and whose serum creatinine has increased substantially (by >50% or ≥266 μmol/L), we recommend discontinuing ACEI/ARB, monitoring renal function, and switching to ARNI after renal function stabilizes or improves, so as to reduce the risk of entering end-stage renal disease. *(1B)*

**Rationale:** A review published in 2021 noted that the sympathetic system was strongly activated in patients with heart failure, and using a RAAS inhibitor alone to dilate efferent arterioles could significantly reduce the hydrostatic pressure in capillaries, resulting in an increase in creatinine. When ARNI was used instead of RAAS inhibitors, efferent and afferent arterioles were dilated synchronously, the renal plasma flow increased, the effective filtration area increased, and the filtration permeability of mesangial cells increased, thus improving eGFR (73). In the PARADIGM-HF study, the risk of increased serum creatinine was significantly lower in the ARNI group than in the enalapril group. A meta-analysis published in ASN in 2020 reported the same benefits of ARNI, as well as a reduction in the risk of elevated serum creatinine by 14% compared with ACEI/ARB (ORR = 0.86; 95% CI = 0.78–0.95, *P* = 0.002) (72).

#### If CKD Is Accompanied by Overhydration That Persists, We Recommend Switching to ARNI

**Suggestion 3.4.5:** For patients with CKD who still exhibit overhydration after treatment with ACEI/ARB, we suggest switching ACEI/ARB to ARNI to improve volume control. *(2C)*

**Rationale:** ARNI not only inhibits the RAAS system, but also inhibits enkephalinase and enhances the natriuretic peptide system. The natriuretic peptide system is a potential target for renal disease treatment, which can promote natriuresis and diuresis, help reduce sodium as well as water retention, and improve volume control (73).

### Can ARNI Directly Replace ACEI/ARB?

**Suggestion 3.5:** For patients with HFrEF not previously treated with ACEI/ARB, ARNI treatment should be the first choice to reduce the risk of cardiovascular death and hospitalization owing to heart failure. *(1B)*

**Rationale:** Subgroup analysis of the PIONEER-HF study confirmed that direct initiation of ARNI had more clinical benefits than switching from ACEI to ARNI. Further, the benefits were consistent among all patients in the high-risk subgroup (Supplementary Table 3). The risk of adverse reactions was similar in both high-risk and low-risk patients. This indicated that, in the former, direct initiation of ARNI during hospitalization had more benefits and was well tolerated (33). The TRANSITION study further confirmed that early initiation of ARNI during hospitalization was safe and effective (31). Therefore, in patients with heart failure, directly initiating ARNI can avoid the treatment delay caused by initial ACEI intake followed by a switch to ARNI. The 2021 US expert consensus on the management of patients with HFrEF states that ARNI should be preferred over ACEI/ARB for patients with HFrEF (74).

## ARNI in Patients with CKD Undergoing Maintenance Dialysis (Hemodialysis and Peritoneal Dialysis)

### What Are the Indications of ARNI in Patients Undergoing Maintenance Dialysis?

**Suggestion 4.1.1:** For patients with heart failure undergoing maintenance dialysis, ARNI is recommended for improving myocardial remodeling, controlling the symptoms of heart failure, protecting residual renal function, and reducing the risk of cardiovascular events. *(1B)*

**Rationale:** Data exist on the use of ARNI in patients with heart failure undergoing maintenance dialysis. A clinical study conducted in hemodialysis patients in China showed that, when ARNI 100 mg BID was used to treat hemodialysis patients with HFrEF or heart failure with midrange ejection fraction, the maximum blood concentration of LBQ657 was within the safe drug concentration range, and ARNI effectively improved their left ventricular ejection fraction (*P* < 0.05) (75). Another retrospective study including 23 patients also confirmed that ARNI could improve the left ventricular ejection fraction and myocardial marker levels in dialysis patients with HFrEF (76). A Chinese study including 21 peritoneal dialysis patients with HFpEF confirmed that ARNI could also significantly improve the symptoms and signs of heart failure and reduce the levels of heart failure markers, while also exhibiting a tendency to improve cardiac function (77).

**Suggestion 4.1.2:** ARNI can be used in patients with hypertension undergoing maintenance dialysis to lower blood pressure, protect cardiac function, and reduce the risk of cardiovascular events. *(2D)*

**Rationale:** Approximately 90% of dialysis patients in China have hypertension (78). The blood pressure of dialysis patients is mostly managed *via* combination therapy (79). However, the blood pressure control rate in patients after antihypertensive treatment is only 25.5% (78). Persistent hypertension in dialysis patients can be correlated not only with left ventricular hypertrophy, but also with ischemic heart disease, heart failure, and cardiovascular mortality (80). Therefore, ARNI can be used in patients with hypertension undergoing maintenance dialysis to lower blood pressure, protect cardiac function, and reduce the risk of cardiovascular events (80). At present, there are limited data on the use of ARNI in dialysis patients with hypertension; therefore, clinical experience should be considered.

### What Precautions Should Be Taken When Using ARNI in Patients Undergoing Maintenance Dialysis?

**Suggestion 4.2:** In patients undergoing maintenance dialysis, we recommend starting ARNI from a low dose of 25–50 mg QD, paying attention to blood pressure during treatment, and adjusting the dose gradually to 100–150 mg QD to BID according to the patient's tolerance (Table 2). *(1C)*

**Rationale:** When used reasonably, ARNI is well tolerated in dialysis patients. In a retrospective study, 23 patients who were switched from ACEI/ARB to ARNI treatment exhibited an average dose of ARNI at the baseline of 90 ± 43 mg/d. After a follow-up period with a median length of 132 days, the dose at the last follow-up was 123 ± 62 mg/d. The most common adverse reaction during treatment was hypotension (SBP <100 mmHg), which usually occurred during or immediately after hemodialysis. During treatment, the dose of ARNI was reduced for approximately 21.7% of patients because of adverse reactions; however, these reactions did not lead to drug discontinuation (76). Therefore, for dialysis patients, we suggest starting with a low dose, monitoring blood pressure during treatment, and gradually adjusting the dose according to tolerance. Research has shown that ARNI at a dose of 100–150 mg BID is safe and tolerable for hemodialysis patients (75, 81).

## Conclusion

When using ARNI in patients with CKD, its dosage and frequency should be determined according to renal function, cardiac function, and the occurrence of hypoproteinemia or abnormal liver function. Whether it is necessary to start with a low dose in patients with moderate and severe renal impairment should be further addressed (Table 3). Although ARNI can be used in combination with SGLT2i for patients with heart failure, its combination with ARB requires further investigation. When used with diuretics, close attention should be paid to adjusting the dosage. When used with spironolactone, amiloride, or potassium salt, close attention should be paid to serum potassium levels, while avoiding ACEI or aliskiren use. ARNI is recommended for non-dialysis patients with CKD exhibiting heart failure or hypertension. In the case of heart failure or poor blood pressure control, we suggest that patients using ACEI/ARB should switch to ARNI. ARNI is recommended to improve cardiac structure and function in maintenance dialysis patients with heart failure. In dialysis patients with hypertension, the efficacy of ARNI requires further investigation.

**Table 3:** Future research suggestions.

ARNI use in patients with hypoproteinemia Hypoalbuminemia is common in patients with CKD. The pharmacokinetic characteristics and ARNI administration approaches in patients with hypoalbuminemia require further investigation.
Safety and efficacy of ARNI combined with ARB There is no clinical research on the combination of ARNI and ARB. Whether the combination of ARNI and ARB can elicit stronger long-term cardiovascular protection and further enhance the urine protein-reducing effect requires further investigation.
ARNI combined with SGLT2i for non-heart failure patients SGLT2i can reduce the risk of heart failure and renal failure as well as cardiovascular and all-cause death. In non-heart failure patients, whether combining ARNI with SGLT2i can have greater clinical benefits requires further investigation.
Do hypertensive patients with moderate and severe renal impairment require a lower loading dose? In patients with moderate and severe renal impairment, the exposure to LBQ657 is significantly increased (by 2–3 times). Whether the loading dose of ARNI should be reduced for these patients requires further investigation.
What is the efficacy and safety of twice-daily ARNI administration in patients with CKD exhibiting hypertension? The incidence of nocturnal hypertension is higher in patients with CKD than in the general population. For patients with CKD and hypertension, the safety and efficacy of twice-daily ARNI administration should be further studied.
What are the application results of ARNI in patients undergoing maintenance dialysis with hypertension? Whether ARNI exhibits better antihypertensive and target organ protective effects than other antihypertension drugs in patients with hypertension undergoing maintenance dialysis requires further investigation.

## Author Contributions

All authors listed have made a substantial, direct, and intellectual contribution to the work and approved it for publication.

## Conflict of Interest

The authors declare that the research was conducted in the absence of any commercial or financial relationships that could be construed as a potential conflict of interest.

## Publisher's Note

All claims expressed in this article are solely those of the authors and do not necessarily represent those of their affiliated organizations, or those of the publisher, the editors and the reviewers. Any product that may be evaluated in this article, or claim that may be made by its manufacturer, is not guaranteed or endorsed by the publisher.
