# Supplementary material for: Application of Angiotensin Receptor–Neprilysin Inhibitor in Chronic Kidney Disease Patients: Chinese Expert Consensus
Source: Front Med (Lausanne). 2022 Jul 19;9:877237. doi: 10.3389/fmed.2022.877237 (PMC9343998; doi:10.3389/fmed.2022.877237)
Supplement: Supplementary file 1 [file Data_Sheet_1.docx]

Supplementary Material

**Supplementary Table 1. Child–Pugh liver function classification**

|  | **1 point** | **2 points** | **3 points** |
| --- | --- | --- | --- |
| Hepatic encephalopathy (classification) | No | Grade 1–2 | Grade 3–4 |
| Ascites | No | Mild | Moderate to severe |
| Total bilirubin (μmol/L) | <34 | 34–51 | >51 |
| Albumin (g/L) | >35 | 28–35 | <28 |
| Prolonged prothrombin time (s) | <4 | 4–6 | >6 |

Note: Grade A (5–6); Grade B (7–9); Grade C (10–15) (Durand F, Valla D. Assessment of the prognosis of cirrhosis: Child–Pugh versus MELD. J Hepatol (2005) 42 Suppl:S100–7.)

**Supplementary Table 2. Criteria for hemodynamic stability according to TRANSITION and PIONEER-HF studies**

| **Criteria for hemodynamic stability** |
| --- |
| Blood pressure has been measured at least three times in the last 6–12 h, with SBP≥100 mmHg |
| Normal blood volume |
| In the last 6–12 h, the dosage of intravenous or oral diuretics has remained unchanged |
| Intravenous vasodilators, intravenous inotropic drugs or vasopressors have been discontinued for at least 6–12 h |

Wachter R, Senni M, Belohlavek J, Straburzynska-Migaj E, Witte KK, Kobalava Z, et al. Initiation of sacubitril/valsartan in haemodynamically stabilised heart failure patients in hospital or early after discharge: Primary results of the randomised TRANSITION study. Eur J Heart Fail (2019) 21:998–1007.

DeVore AD, Braunwald E, Morrow DA, Duffy CI, Ambrosy AP, Chakraborty H, et al. Initiation of angiotensin-neprilysin inhibition after acute decompensated heart failure: Secondary analysis of the open-label extension of the Pioneer-HF trial. JAMA Cardiol (2020) 5:202–7.

**Supplementary Table 3. Definition of high-risk subgroup according to the PIONEER-HF study**

| **High-risk group criteria** |
| --- |
| Patients with baseline SBP ≤118 mmHg |
| Baseline NT-proBNP >2,701 pg/mL |
| Estimated glomerular filtration rate <60 mL/min/1.73 m^2^ |
| Hospitalized due to heart failure ≥1 time in the past year |
| Patients admitted to ICU |
| Patients who used cardiotonic drugs during hospitalization |
| Patients with severe congestion determined by a congestion score ≥ 4 |

Berg DD, Samsky MD, Velazquez EJ, Duffy CI, Gurmu Y, Braunwald E, et al. Efficacy and Safety of Sacubitril/Valsartan in High-Risk Patients in the PIONEER-HF Trial. Circ Heart Fail. 2021;14(2):e007034.

**Supplementary Table 4. Criteria for calculating the congestion score according to the PIONEER-HF study**

| **Congestion score is the sum of severity scores of the following criteria:** |
| --- |
| **O**rthopnea: not present = 0, seldom = 1, frequent = 2, and continuous = 3 |
| **R**ales: not present = 0, basilar = 1, and > 1/3 of lung fields = 2 |
| **P**eripheral edema: not present = 0, trace, feet/ankle = 1, leg/thigh = 2, sacrum = 3 |

Berg DD, Samsky MD, Velazquez EJ, Duffy CI, Gurmu Y, Braunwald E, et al. Efficacy and Safety of Sacubitril/Valsartan in High-Risk Patients in the PIONEER-HF Trial. Circ Heart Fail. 2021;14(2):e007034.

**Supplementary Table 5. Diagnosis criteria for mild, moderate, and severe renal impairment**

| **Diagnosis criteria for mild, moderate, and severe renal impairment** |
| --- |
| **M**ild renal impairment: eGFR 60–90 mL/min/1.73 m^2^ |
| **M**oderate renal impairment: eGFR 30–60 mL/min/1.73 m^2^ |
| **S**evere renal impairment: eGFR <30 ml/min/1.73 m^2^ |

eGFR: estimated glomerular filtration rate
